# One patient with synchronous multiple primary colon cancers which is easy to miss: a case report

**DOI:** 10.3389/fsurg.2026.1939216

**Published:** 2026-09-15

**Authors:** Xinmin Bao, Haoru Liu, Ming Wu, Xianwei Liu

**Affiliations:** 1 Department of General Surgery, Jiujiang City Key Laboratory of Cell Therapy, Jiujiang No.1 People’s Hospital, Jiujiang, Jiangxi, China; 2 Department of Pathology, Jiujiang City Key Laboratory of Cell Therapy, Jiujiang No.1 People’s Hospital, Jiujiang, Jiangxi, China; 3 Department of Image Center, Jiujiang City Key Laboratory of Cell Therapy, Jiujiang No.1 People’s Hospital, Jiujiang, Jiangxi, China

**Keywords:** CT, endoscopic obstruction, missed diagnosis, multiple primary colon cancer, operation

## Abstract

**Background:**

Multiple primary colon cancer is a relatively rare type of colon cancer (CC), and it is even rarer to have endoscopic obstruction.

**Case summary:**

A 70-year-old female patient was previously diagnosed with sigmoid colon cancer accompanied by endoscopic obstruction at an external hospital. Preoperative abdominal enhanced CT in our hospital revealed one segmental bowel wall thickening with intussusception, and two separate thickened lesions in the transverse colon. During the operation, it was found that the intestinal wall of sigmoid colon, the transverse colon near splenic flexure and the ascending colon were obviously thickened and stiff, and the lymph nodes at the root of inferior mesenteric artery, the ileocolic artery and middle colon artery were swollen. Finally, we performed laparoscopic sigmoidectomy, left hemicolectomy, and right hemicolectomy with mesenteric lymph node dissection and ileorectal anastomosis. Postoperative pathological examination confirmed that adenocarcinoma existed in sigmoid colon, transverse colon and ascending colon.

**Conclusion:**

Intraoperative comprehensive exploration is critical for the management of obstructive colon cancer. For patients with incomplete preoperative colonoscopy caused by tumor-induced luminal obstruction, meticulous intraoperative assessment is essential to avoid missed diagnosis of synchronous multiple CC and optimize long-term oncological outcomes.

## Introduction

Multiple primary colon cancer (MPCC) is defined as two or more primary colon cancers (CC) occurring in a single patient (1, 2). As a relatively rare type of CC, it is mainly divided into synchronous colon cancer (SCC) and metachronous colon cancer (MCC) (3). At present, there is no unified standard for SCC and MCC. Generally speaking, SCC refers to the primary CC detected simultaneously or within 6 months after the first CRC was diagnosed, while MCC means that the interval of diagnosis is more than 6 months (4). Patients with familial adenomatous polyposis (FAP), Lynch syndrome (LS) and inflammatory bowel disease (IBS) are generally considered to be at higher risk of SCC (5). Compared with isolated CC, SCC is more common in the proximal colon of elderly men, with mucinous adenocarcinoma, advanced TNM stage and microsatellite instability-high (MSI-H) status (6). Most studies reported that the prognosis of SCC patients was worse than that of patients with isolated CC (6–8). Endoscopic obstruction is a common clinical manifestation of advanced CC,caused by tumor-induced intestinal stenosis that prevents complete endoscopic evaluation of the proximal colon segment (9). This diagnostic limitation frequently leads to inadvertent missed detection of proximal colonic lesions in routine clinical practice (10). Herein, we report a case of complete endoscopic obstruction caused by a primary sigmoid colon adenocarcinoma. Preoperative evaluation failed to visualize the proximal colon, and abdominal enhanced CT only suggested tumorous lesions in the sigmoid and transverse colon. However, thorough intraoperative exploration finally identified multiple synchronous and occult malignancies involving the transverse and ascending colon.

## Case

**Figure 1:**
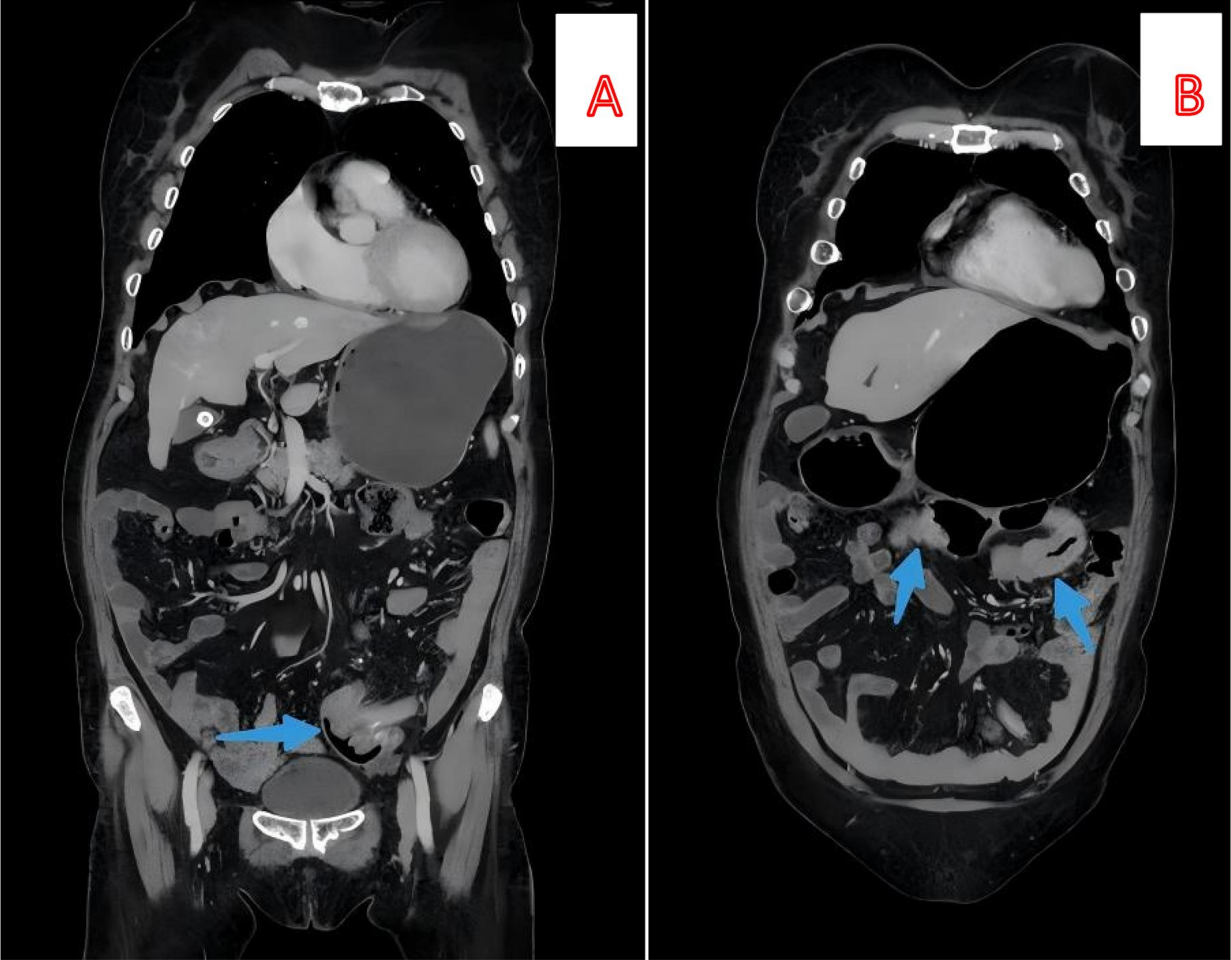
Preoperative enhanced CT images of sigmoid colon tumor **(A)** and transverse colon tumor **(B)** the area indicated by the blue arrow is the thickened part of the colon wall that is considered to be a tumor.

A 70-year-old female patient suffered from paroxysmal dull pain in the upper abdomen with a change of defecation habits for more than 3 months. The enteroscopy in the outer hospital showed that the sigmoid colon tumor was accompanied by intestinal stenosis, and the endoscope could not pass (high-definition colonoscopy images could not be obtained). Biopsy results confirmed moderately differentiated adenocarcinoma. The patient had a history of type 2 diabetes before, and her blood sugar was well controlled by taking metformin and glimepiride regularly. There is no special medical history in the family. The patient's vital signs are stable, and her abdomen was flat and soft, without tenderness, rebound pain and muscle tension. The moving dullness was negative and the bowel sounds were normal. No abnormality was found in digital examination of anus. It was found that hemoglobin was 82 g/L, CEA was 79.2 ug/L and CA-199 was 46.30 u/mL. Preoperative chest computed tomography (CT) showed no tumor lesions, and abdominal enhanced CT identified sigmoid colon wall thickening accompanied by intussusception (Figure 1A), and two independent thickened segments in the transverse colon (Figure 1B). After conservative treatment such as fasting, nutritional support, enema and paraffin oil lubrication, enteroscopy still failed to pass the sigmoid colon tumor.

**Figure 2:**
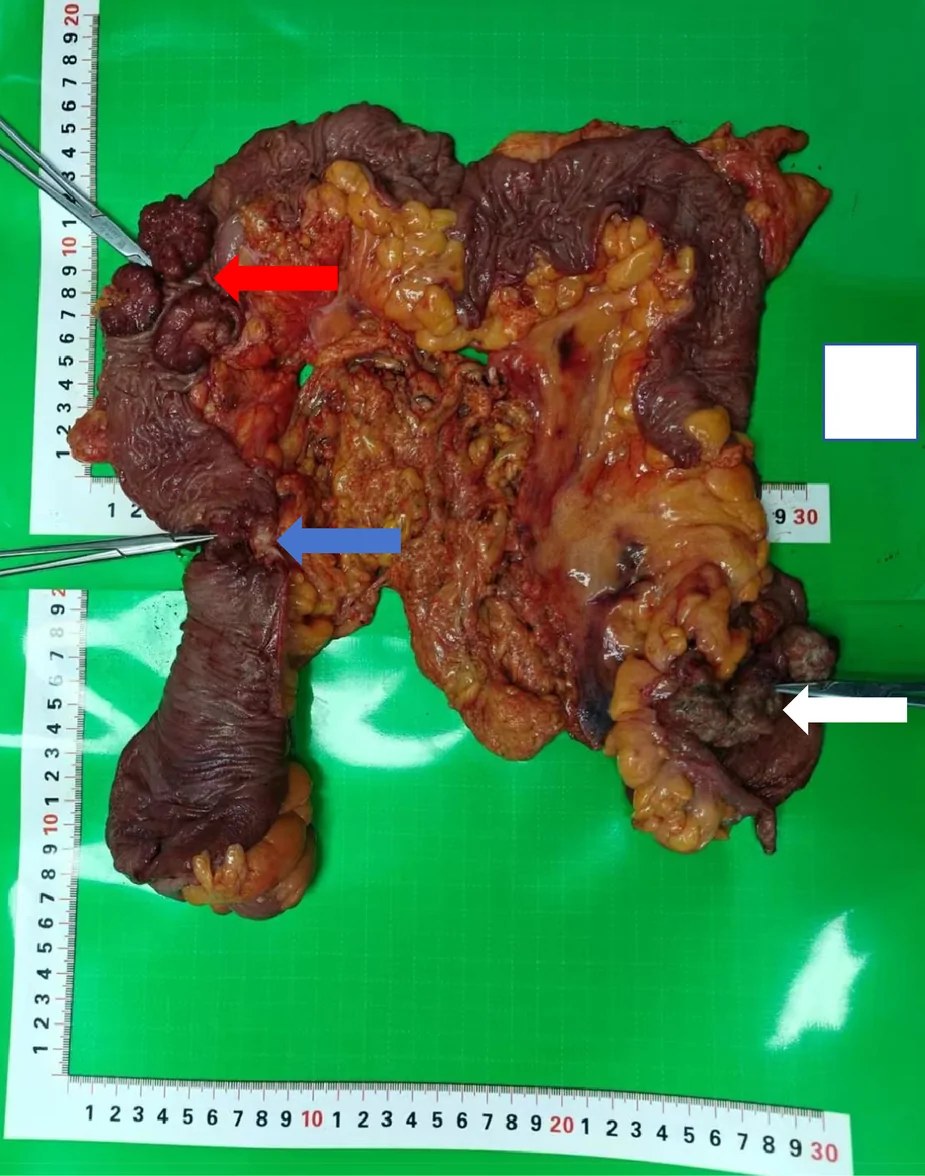
The colon specimen. The blue arrow indicates sigmoid colon tumor, the red arrow indicates transverse colon tumor and the white arrow indicates ascending colon tumor, R stands for direction-right.

**Figure 3:**
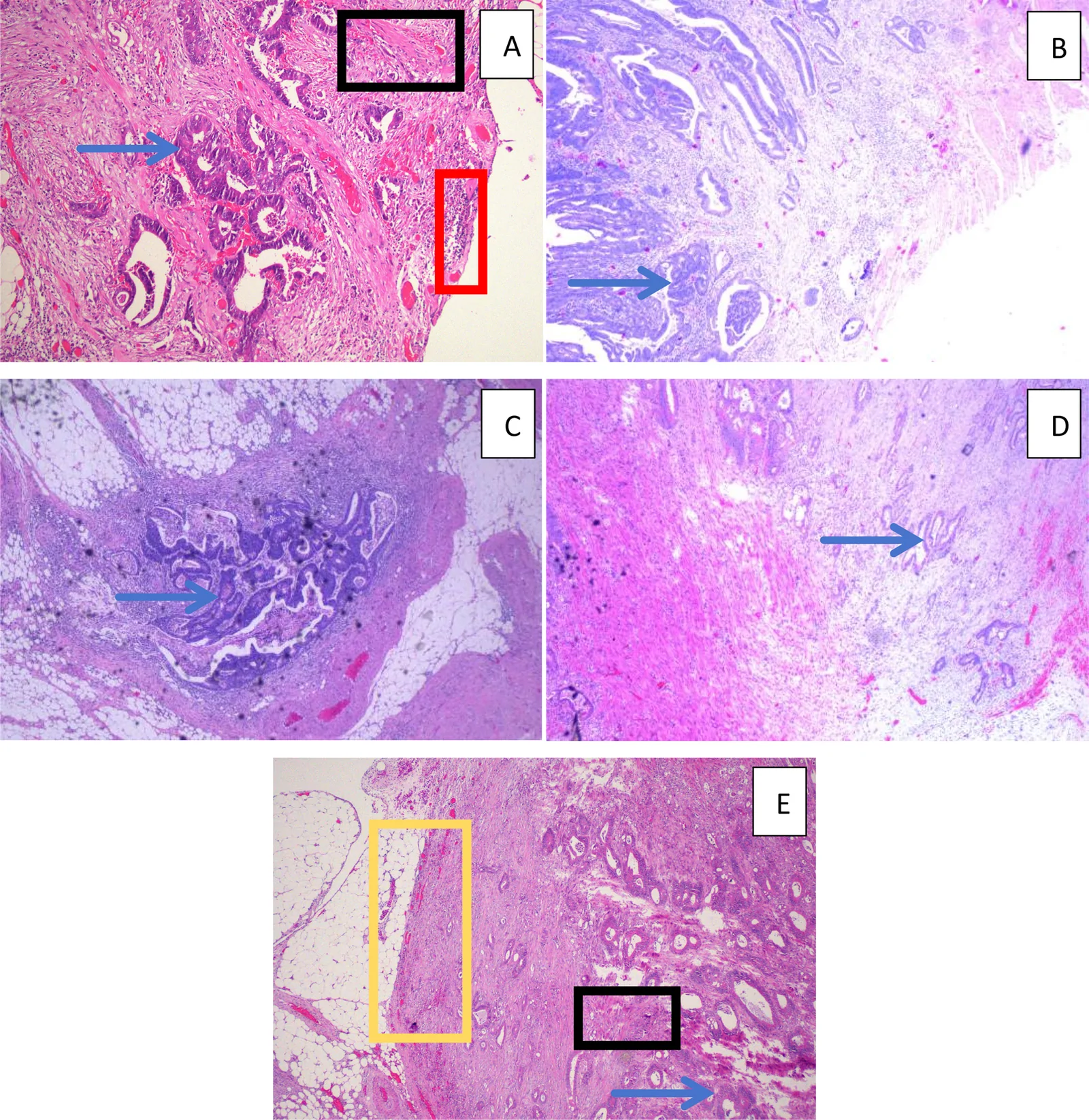
Pathological images of the 5 tumors (HE, × 100). **(A)** The sigmoid colon tumor; **(B)** The ascending colon tumor; **(C)** Tumor 1 of the transverse colon; **(D)** Tumor 2 of the transverse colon; **(E)** Tumor 3 of the transverse colon. The blue arrows are all adenocarcinoma, the black box is the nerve invasion area, the red box is the serous breakthrough area, and the yellow box is the serous invasion area.

For this patient, we routinely conducted discussions with multidisciplinary teams (including imaging center, pathology department, oncology department, anesthesia department, nutrition department, etc.). The final diagnosis was sigmoid adenocarcinoma with endoscopic obstruction (cT3N + M0), and transverse colon malignant tumor was not ruled out. Therefore, the recommended surgery is laparoscopic sigmoidectomy, left hemicolectomy and mesenteric lymph node dissection, the specific surgical plan is determined according to the intraoperative exploration. After transfusion of red blood cells to improve anemia, we excluded the contraindications of surgery and performed laparoscopic surgery on the patient. During the operation, it was found that the intestinal wall of sigmoid colon, the transverse colon near splenic flexure and the ascending colon were obviously thickened and stiff, and the lymph nodes at the root of inferior mesenteric artery, the ileocolic artery and middle colon artery were swollen. Finally, laparoscopic combined sigmoidectomy, left hemicolectomy, right hemicolectomy, mesenteric lymph node dissection, and ileorectal anastomosis were subsequently performed. The colon specimen is shown in Figure 2. After intussusception reduction and longitudinal opening of the resected colon, we found that one tumor was in the sigmoid colon, one tumor was in the ascending colon and three tumors were in the transverse colon. We reviewed the preoperative abdominal CT, and found no abnormality in the ascending colon. Postoperative pathology showed that the sigmoid colon tumor was a moderately differentiated adenocarcinoma, which penetrated the serosa and showed invasion of blood vessels and nerves (Figure 3A). The tumor of ascending colon was a moderately differentiated adenocarcinoma and invaded the muscular layer (Figure 3B). Tumor 1 of the transverse colon was moderately differentiated adenocarcinoma, which penetrated the muscular layer and invaded the serosa (Figure 3C). Tumor 2 of the transverse colon was moderately differentiated adenocarcinoma and invaded the muscular layer (Figure 3D). And tumor 3 of the transverse colon was moderately differentiated adenocarcinoma, which penetrated the muscular layer and invaded the serosa, showing nerve invasion (Figure 3E). There were 7 lymph nodes around sigmoid colon without metastasis; 11 lymph nodes around the left colon without metastasis; 7 lymph nodes around the right colon, and cancer cells were found in one lymph node. According to the TNM staging system and NCCN guidelines, individual tumor stages were defined as T4N0M0 (sigmoid colon), T2N1M0 (ascending colon), T3N0M0 (transverse colon tumor 1), T2N0M0 (transverse colon tumor 2), and T3N0M0 (transverse colon tumor 3). The overall pathological stage was determined as T4N1M0 (stage IIIB) based on the most advanced tumor and nodal status. Immunohistochemical results showed that PMS 2 (+), MSH6 (+), MSH 2 (+), MLH 1 (+). Although the sigmoid colon tumor is not large, it not only penetrates the serosa and grows constrictively, but also is accompanied by intussusception. This also explains why it leads to endoscopic obstruction. Combined with the patient's family history, pathological findings, mismatch repair immunohistochemistry and enterotomy test results, FAP, LS and IBS were excluded. 10 days after the operation, the patient recovered and was discharged. On the 21st day after the operation, the patient returned to the first cycle of Xelox chemotherapy, and then continued for 8 cycles. No obvious evidence of recurrence and metastasis was found in 2 years follow-up after chemotherapy (Table 1).

**Table 1:** Timeline of the patient's clinical course.

Time point	Clinical event	Key findings/Intervention
Index presentation	Initial symptoms	Paroxysmal dull pain in the upper abdomen with a change of defecation habits for more than 3 months
Outer hospitalwork-up	Colonoscopy	The sigmoid colon tumor was accompanied by intestinal stenosis, and the endoscope could not pass
Admission work-up	Abdominal CT	The intestinal wall of the sigmoid colon was thickened with intussusception at one place and of the transverse colon was thickened at two places
Hospitalization	Surgical treatment	Laparoscopic sigmoidectomy, left hemicolectomy, right hemicolectomy, mesenteric lymph node dissection, ileum-rectum anastomosis
Post-operative	Pathological examination	Adenocarcinoma existed in sigmoid colon, transverse colon and ascending colon
Post-operative period	Adjuvant chemotherapy	Xelox, 8 cycles
Subsequent visits	Follow-up surveillance	2 years, no obvious signs of recurrence and metastasis were found (examination items include colonoscopy, abdominal CT, CEA, etc.)

## Discussion

Endoscopic obstruction is mainly lumen stenosis caused by tumor growth, which usually leads to systemic complications (9). This is a relatively common clinical problem, and some patients even need emergency surgery (11–13). Endoscopic obstruction caused by colon malignant tumor, which were finally confirmed as multiple primary colon cancers, has also been found from time to time (14, 15). However, our case is extremely rare, as the patient presented with sigmoid tumor-related complete endoscopic obstruction that prohibited preoperative evaluation of the entire proximal colon. Repeated colonoscopy after conservative treatment still failed to bypass the stenotic lesion. Preoperative enhanced CT only suggested sigmoid malignancy and two suspicious transverse colon lesions, while intraoperative exploration unexpectedly identified a total of five synchronous primary tumors involving the sigmoid, transverse, and ascending colon.

The sensitivity of CT to stage Ⅰ and Ⅱ of CC is limited (16),primarily due to subtle bowel wall thickening, intestinal peristalsis artifacts, volume averaging effects, and variable tumor growth patterns. Although many studies showed that the combined artificial intelligence technology can effectively improve the diagnostic accuracy (17–19), it can not be widely promoted. In this case, the missed ascending colon tumor only invaded the muscularis propria with minimal morphological changes, rendering it undetectable on preoperative CT scans, which explains the preoperative diagnostic discrepancy.

For patients with incomplete preoperative colonoscopy due to tumor obstruction, repeat endoscopic examination after conservative decompression is the standard strategy to rule out proximal synchronous lesions (20). Unfortunately, this approach was ineffective in our case. Intraoperative colonoscopy via the stenotic segment represents a potential solution to avoid missed diagnosis, though this technique remains controversial (21). Intraoperative endoscopy carries increased risks of abdominal and incision infection due to inadequate preoperative bowel preparation, requiring further large-scale clinical validation. Additionally, although Positron Emission Tomography-CT (PET-CT) (22) can serve as effective supplementary imaging modalities for detecting occult colonic malignancies, the high cost limits routine clinical application. Furthermore, these modalities do not achieve 100% diagnostic sensitivity for colonic tumors.

Therefore, we recommend that for patients with obstructive colon cancer in whom complete preoperative colonoscopy is unfeasible, meticulous preoperative abdominal evaluation with contrast-enhanced CT is essential to minimize missed lesions. PET-CT can be selectively applied in high-risk scenarios. Most importantly, comprehensive intraoperative exploration and feasible intraoperative colonoscopy are strongly recommended. Inadequate intraoperative assessment may lead to undiagnosed synchronous tumors, resulting in secondary surgery with increased technical difficulty and postoperative risks.

Currently, there is no consensus or clearly stated in current guidelines on the diagnosis and treatment of MPCC (3). Generally speaking, the choice of surgical methods mainly depends on the position of the tumor in the colon (3, 23, 24). When the tumors are located in a single region or adjacent part of the colon, standard colectomy or adjacent part extended colectomy is selected. When the tumors are located in several different areas, segmental resection or subtotal resection of colon can be chosen. The operation we performed on the patients conforms to the surgical methods recommended by mainstream academic circles. Most studies reported that the prognosis of SCC patients is worse than that of patients with isolated CC, which may be related to the more advanced tumor stage, larger tumor load, wider resection range and more postoperative complications. Fortunately, our patient achieved favorable short-term oncological outcomes with no recurrence or metastasis during 2 years of follow-up after radical surgery and standardized adjuvant chemotherapy, with long-term surveillance ongoing.

## Conclusion

Intraoperative comprehensive exploration is critical for the management of obstructive colon cancer. For patients with incomplete preoperative colonoscopy caused by tumor-induced luminal obstruction, meticulous intraoperative assessment is essential to avoid missed diagnosis of synchronous multiple CC and optimize long-term oncological outcomes.

## Data Availability

The raw data supporting the conclusions of this article will be made available by the authors, without undue reservation.
